# Apoptotic endothelial cells release small extracellular vesicles loaded with immunostimulatory viral-like RNAs

**DOI:** 10.1038/s41598-019-43591-y

**Published:** 2019-05-10

**Authors:** Marie-Pierre Hardy, Éric Audemard, Francis Migneault, Albert Feghaly, Sylvie Brochu, Patrick Gendron, Éric Boilard, François Major, Mélanie Dieudé, Marie-Josée Hébert, Claude Perreault

**Affiliations:** 1 Institute for Research in Immunology and Cancer, Université de Montréal, Montreal, QC H3C 3J7 Canada; 2 Canadian National Transplant Research Program, Edmonton, Alberta T6G 2E1 Canada; 3 Research Centre, Centre Hospitalier de l′Université de Montréal (CRCHUM), Montreal, QC H2X 0A9 Canada; 4 Centre de Recherche du Centre Hospitalier Universitaire de Québec, Faculté de Médecine de l′Université Laval, Québec, Québec Canada; 5 Department of Computer Science and Operations Research, Université de Montréal, Montreal, QC H3C 3J7 Canada; 6 Department of Biochemistry, Faculty of Medicine, Université de Montréal, Montreal, QC H3C 3J7 Canada; 7 Department of Medicine, Université de Montréal, Montreal, QC H3C 3J7 Canada

**Keywords:** Transcriptomics, RNA, Pattern recognition receptors, Systems analysis

## Abstract

Endothelial cells have multifaceted interactions with the immune system, both as initiators and targets of immune responses. *In vivo*, apoptotic endothelial cells release two types of extracellular vesicles upon caspase-3 activation: apoptotic bodies and exosome-like nanovesicles (ApoExos). Only ApoExos are immunogenic: their injection causes inflammation and autoimmunity in mice. Based on deep sequencing of total RNA, we report that apoptotic bodies and ApoExos are loaded with divergent RNA cargos that are not released by healthy endothelial cells. Apoptotic bodies, like endothelial cells, contain mainly ribosomal RNA whereas ApoExos essentially contain non-ribosomal non-coding RNAs. Endogenous retroelements, bearing viral-like features, represented half of total ApoExos RNA content. ApoExos also contained several copies of unedited *Alu* repeats and large amounts of non-coding RNAs with a demonstrated role in autoimmunity such as U1 RNA and Y RNA. Moreover, ApoExos RNAs had a unique nucleotide composition and secondary structure characterized by strong enrichment in U-rich motifs and unstably folded RNAs. Globally, ApoExos were therefore loaded with RNAs that can stimulate a variety of RIG-I-like receptors and endosomal TLRs. Hence, apoptotic endothelial cells selectively sort in ApoExos a diversified repertoire of immunostimulatory “self RNAs” that are tailor-made for initiation of innate immune responses and autoimmunity.

## Introduction

Endothelial cells have multifaceted interactions with the immune system, both as initiators and targets of immune responses. The endothelium can recruit immune cells to sites of inflammation or injury, trigger innate immune responses and present antigens to B and T lymphocytes^1^. Endothelial cells are also the targets of immune processes leading to atherosclerosis, hypertension, microangiopathy and vasculitis^2–5^. Pleiotropic interactions between endothelial and immune cells find particularly vivid illustration in the context of transplantation, where endothelial cells act as initiators and critical targets of graft rejection and graft-versus-host disease (GVHD)^6,7^. Indeed, antibodies targeting the vasculature of solid allografts are associated with poor outcome in solid organ transplantation, and endothelial damage contributes to the severity of GVHD^7,8^.

Extracellular vesicles (EVs) are now recognized as masters of intercellular communication and most of their functions are in the regulation of immune responses^9–12^. Notably, EVs released by allogeneic immune cells were shown to trigger proinflammatory T cell responses in different models of solid organ transplantation^11,12^. Furthermore, we recently reported that exosome-like nanovesicles released by apoptotic mouse endothelial cells accelerate rejection of vascular grafts^13,14^. Consistent with the deleterious effect of autoantibodies in graft rejection and GVHD^15,16^, we observed that EVs from apoptotic mouse endothelial cells induced production of anti-nuclear antibodies and autoantibodies against the LG3 fragment of perlecan^13^. Autoantibody production was not associated with increased levels of anti-MHC antibodies, but correlated with increased graft infiltration by B and T cells. Notably, none of the effects of apoptotic exosome-like vesicles were observed following injection of apoptotic bodies^13^. Hence, apoptotic exosome-like vesicles represent a novel and immunogenic component of the paracrine apoptotic response. Moreover, our data allow for the development of a model that integrates and explains numerous reports linking tissue injury to autoantibody production and graft rejection or GVHD^15^. A key question is how apoptotic exosome-like vesicles (ApoExos) may trigger an autoimmune cascade. An attractive explanation is that these vesicles would contain danger associated molecular patterns (DAMP) that bind to pattern recognition receptors (PRRs) on innate immune cells. In our quest to identify such DAMPs, we elected to analyze the transcriptome of ApoExos. This choice was based on two premises. First, nucleic acids are ligands for the most diversified repertoire of PRRs, which includes toll-like receptors (TLRs), RIG-I-like receptors (RLRs) and members of the cGAS-STING pathway^17^. Second, EVs have been shown to carry functional RNAs that can be sensed by PRRs^9,18^; the nature of these RNAs is cell type dependent and dictated by the metabolic state of the cells^19,20^.

In the present work, we sequenced the whole RNA content of i) apoptotic human umbilical vein endothelial cells (HUVECs) and ii) of the two types of EVs released by these apoptotic HUVECs: apoptotic bodies and ApoExos^13^. Our results revealed that ApoExos have a distinct transcriptomic profile and carry non-coding RNA sequences exhibiting immunostimulatory potential, including mitochondrial transfer RNAs, U1 small nuclear RNA, and pathogen-like endogenous retroelements. Moreover, we show that RNA editing by adenosine deaminases acting on RNA, an important mechanism in self vs. non-self-discrimination of nucleic acids, was reduced in ApoExos. We also observed in ApoExos a dramatic enrichment for Poly-U, AU- and GU-rich motifs, known to be TLR7 and TLR8 agonists. Finally, RNA structure modeling revealed that ApoExos displayed a higher abundance of unstably folded RNA sequences which are more prone to generate single-stranded structures, the preferred ligands of endosomal TLRs. Overall, our work demonstrates that apoptotic endothelial cells release EVs loaded with RNAs which are recognized by RLRs and endosomal TLRs (TLR3, TLR7 and TLR8), and therefore have the ability to elicit innate immune responses.

## Results

### ApoExos are enriched in RNAs derived from non-exonic genomic regions

**Figure 1:**
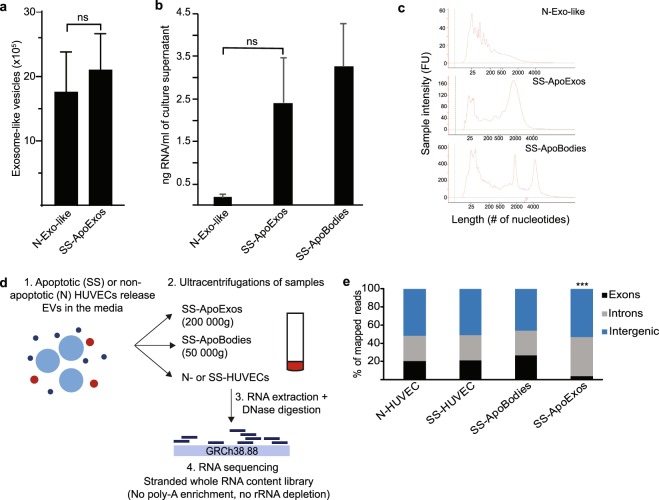
Features of EVs produced by normal and apoptotic HUVECs. (**a**) Normal and apoptotic HUVECs generate similar amounts of exosome-like vesicles. Small particle flow cytometry analysis of CMFDA stained exosome-like vesicles released by HUVECs cultured in standard (N) or serum-starved (SS) condition and isolated from the same volume of culture media (two-tailed unpaired T test, p = 0.7, n = 4). (**b**) Vesicles released by apoptotic HUVECs contain more RNA. Bioanalyzer quantification of RNA extracted from EVs released by SS-HUVECs (SS-ApoExos and SS-ApoBodies) vs. N-HUVECs (N-exo-like vesicles); two-tailed unpaired T test, p = 0.098, n = 2. (**c**) Vesicles released by N-HUVECs contain small or fragmented RNAs. Bioanalyzer profiles of RNA extracted from EVs released by SS-HUVECs (ApoExos and ApoBodies) vs. N-HUVECs (N-exo-like vesicles). (**d**) Workflow for generation and isolation of EVs and for RNA extraction and sequencing. See Methods for details. (**e**) Distribution of RNA-Seq reads in ApoExos. STAR mapped RNA-Seq reads were categorized as exonic, intronic or intergenic using RSeQC 2.6.3. ApoExos contain significantly more intronic sequences and less exonic sequences than apoptotic bodies (***Two-tailed Fisher exact test, p = 1.1 × 10^−5^) and HUVECs (**Two-tailed Fisher exact test, p = 3 × 10^−3^).

Injection of syngeneic endothelial ApoExos, but not apoptotic bodies, induces production of autoantibodies and accelerates rejection of vascular grafts^13,15^ Likewise, injection of these EVs increased the severity of GVHD in mice (Supplemental Fig. 1). We therefore postulated that if endothelial ApoExos contained immunostimulatory RNAs (DAMPs), these DAMPs should be present in much higher amounts in ApoExos than in apoptotic bodies. As in previous studies, stress/apoptosis of endothelial cells was induced by serum starvation^13,15^. Vesicles were isolated from HUVECs cultured in vesicles-free standard media (N) or after induction of apoptosis by 4 h serum starvation (SS). Quality control of the obtained vesicles confirmed that, as previously reported^13^, ApoExos contained elevated levels of LG3 (Supplemental Fig. 2a) as well as high caspase-like proteasome activity (Supplemental Fig. 2b) compared to apoptotic bodies. After staining of EVs with 5-chloromethylfluorescein diacetate, flow cytometry analysis showed that while only SS-HUVECs produced apoptotic bodies^13^, N-HUVECs and SS-HUVECs yielded similar amounts of exosome-like vesicles (Fig. 1a). Apoptotic bodies and ApoExos contained substantial amounts of short and long RNAs (Fig. 1b). By contrast, exosome-like EVs released by N-HUVECs contained less RNAs (Fig. 1b), which were of small size and looked more fragmented on the bioanalyzer profile (Fig. 1c), preventing us from generating transcriptomic libraries. Hence, we performed in-depth analyses of the RNA cargo of ApoExos and apoptotic bodies using N-HUVECs and SS-HUVECs as controls in order to evaluate enrichment of RNA species in EVs.

We did not use poly-A capture or ribosomal RNA depletion to isolate RNA because we considered that it was crucial in our RNA-Seq analyses to capture all RNAs present in EVs. Moreover, poly-A capture is appropriate for mRNAs coded by classic exons, but is inadequate for sequencing of intronic and intergenic RNAs including endogenous retroelements which are particularly immunostimulatory^21–25^. We therefore sequenced all RNAs extracted from two biological replicates of N-HUVECs, SS-HUVECs, apoptotic bodies and ApoExos (Fig. 1d). Aligned reads where then categorized as exonic, intronic or intergenic. The salient finding was that the proportion of RNAs coded by exons was decreased by 5- to 7-fold in ApoExos relative to HUVECs and apoptotic bodies (Fig. 1e). This was the first clue that ApoExos have a peculiar RNA cargo.

### ApoExos contain mainly non-ribosomal non-coding RNAs

**Figure 2:**
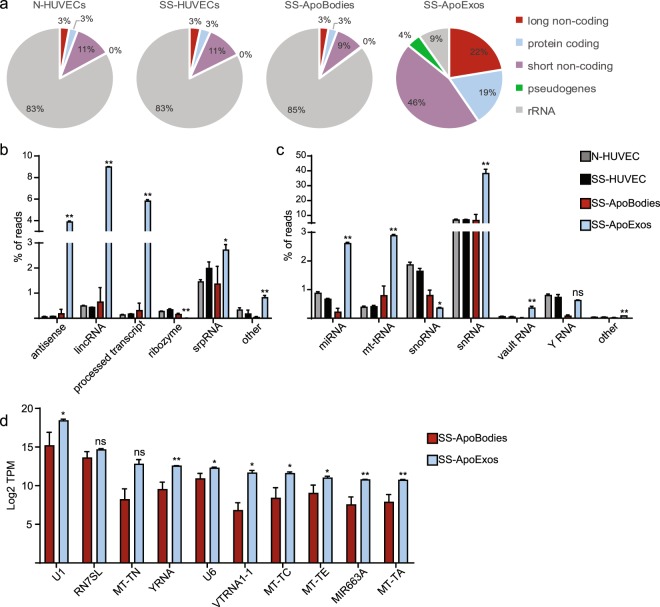
ApoExos contain mainly non-ribosomal non-coding RNAs. (**a**) ApoExos are enriched in non-coding transcripts. RNA-Seq reads were quantified and pseudo-aligned on human Ensembl reference transcriptome using Kallisto. Transcripts were then classified according to their biotype. Pie charts represent the mean proportions of each transcript biotype (n = 2). rRNA: ribosomal RNA. (**b**,**c**) ApoExos are enriched in non-coding transcripts. Detailed distribution of long (**b**) and short (**c**) non-coding RNA transcripts subtypes (Two-tailed unpaired T test, *p ≤ 0.05, **p ≤ 0.01, n = 2). LincRNA: long intergenic non-coding RNA, miRNA: microRNA, mt-tRNA: mitochondrial transfer RNA, snoRNA: small nucleolar RNA, snRNA: small nuclear RNA, srpRNA: signal recognition particle RNA. (**d**) Top ten non-ribosomal non-coding RNAs in ApoExos. Relative abundance in ApoExos and apoptotic bodies expressed as log2 transcripts per million (TPM). (Two-tailed unpaired T test, *p ≤ 0.1, **p ≤ 0.05, n = 2).

RNA-Seq reads were quantified and pseudo-aligned on human Ensembl reference transcriptome using Kallisto^26^, then classified according to their transcript biotype using Ensembl annotation. This analysis uncovered huge differences in RNA types found in ApoExos compared to other samples (Fig. 2a). For example, ribosomal RNAs accounted for 83–85% of RNAs in HUVECs and apoptotic bodies, but only 9% in ApoExos. Moreover, non-coding RNAs (short, long and pseudogenes) represented 72% of RNAs in ApoExos, but only 11–14% of RNAs in HUVECs and apoptotic bodies. Long non-coding RNAs enriched in ApoExos belonged to three main categories: antisense RNAs, long intergenic non-coding RNAs and processed RNAs (Fig. 2b). Enrichment for four types of short non-coding RNAs was conspicuous in ApoExos: microRNAs, mitochondrial transfer RNAs, vault RNAs, and small nuclear RNAs (Fig. 2c). Non-coding RNAs, particularly the short ones, have been shown to stimulate innate immune receptors^10,27^. For example, microRNAs activate TLR7 and TLR8, and mitochondrial transfer RNAs stimulate protein kinase R^28,29^. Vault RNAs are produced by RNA polymerase III, and RNA polymerase III transcripts 5′-triphosphate motif triggers the Retinoic Acid Inducible Gene 1 (RIG-I), the prototypic RLR^20^. Non-ribosomal non-coding RNAs with the highest abundance in ApoExos (Fig. 2d) could potentially be used as markers of endothelial damage. Notably, the transcript which was at the top of this hierarchy was the small nuclear RNA U1 which represented one third of ApoExos annotated transcripts (Fig. 2d). Upon apoptosis, U1 RNA is unshielded by spliceosomal proteins and triggers a wide variety of PRRs^30–32^. U1 RNA has also been shown to act as an adjuvant triggering auto-antibody generation in autoimmune disorders such as systemic lupus erythematosus and mixed connective tissue disease^33^.

### HUVECs-derived ApoExos carry viral-like RNAs

Endogenous retroelements (EREs) represent about 43% of the human genome and belong to three main classes: long terminal repeats (LTRs), long interspersed nuclear elements (LINEs) and short interspersed nuclear elements (SINEs)^23,34^. EREs undergo increased transcriptional activity upon stress conditions, and their transcripts are highly immunogenic^23,35–37^. Indeed, LINEs and SINEs are polymerase III transcripts bearing a 5′-triphosphate motif, a well-characterized RIG-I ligand^20^. In addition, LTRs can undergo bidirectional transcription resulting in double-stranded RNA (dsRNA) secondary structures that trigger TLR3 and the RLR Melanoma Differentiation-Associated protein 5 (MDA5)^35,38–40^. Other less well characterized ‟repetitive sequences” (repeats) share features of EREs^41^. However, it is common practice to filter out EREs and repetitive transcripts in order to simplify transcriptomic analyses. This bias is propagated by alignment tools such as BLAST that mask ‟low complexity and repetitive regions” as a default option^24^.

**Figure 3:**
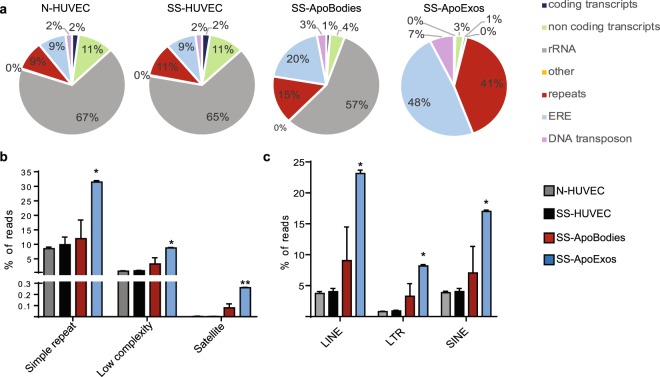
HUVECs-derived ApoExos are loaded with transcripts coded by EREs and repetitive sequences. (**a**) RNA-Seq reads were quantified and pseudo-aligned on human Ensembl reference transcriptome combined to repeat masker annotations using Kallisto. Transcripts were then classified according to their origin. Pie charts represent the mean proportions of each RNA sequence type (n = 2). (**b**,**c**) ApoExos are enriched in repeats and EREs. Detailed distribution of repeats (**b**) and EREs (**c**) RNA sequences across samples (Two-tailed unpaired T test, *p ≤ 0.05, **p ≤ 0.01, n = 2).

To further evaluate the origin of non-coding RNAs found in ApoExos, we therefore quantified RNA sequences with Kallisto and, as a reference index, we combined both Ensembl annotated transcripts and all annotations from the repeat masker database (http://www.repeatmasker.org/). Again, ApoExos presented a very distinct profile (Fig. 3a). The salient finding was that the proportion of RNA-Seq reads coded by EREs and repeats ranged from 18 to 35% in HUVECs and apoptotic bodies, but reached 89% in ApoExos (Fig. 3a). The abundance of all subclasses of EREs and repetitive sequences was increased in ApoExos (Fig. 3b,c). Hence, ApoExos contain an enormous amount of diversified viral-like RNAs that could trigger autoimmunity.

### ApoExos therefore contain large amounts of unedited *Alu* sequences

**Figure 4:**
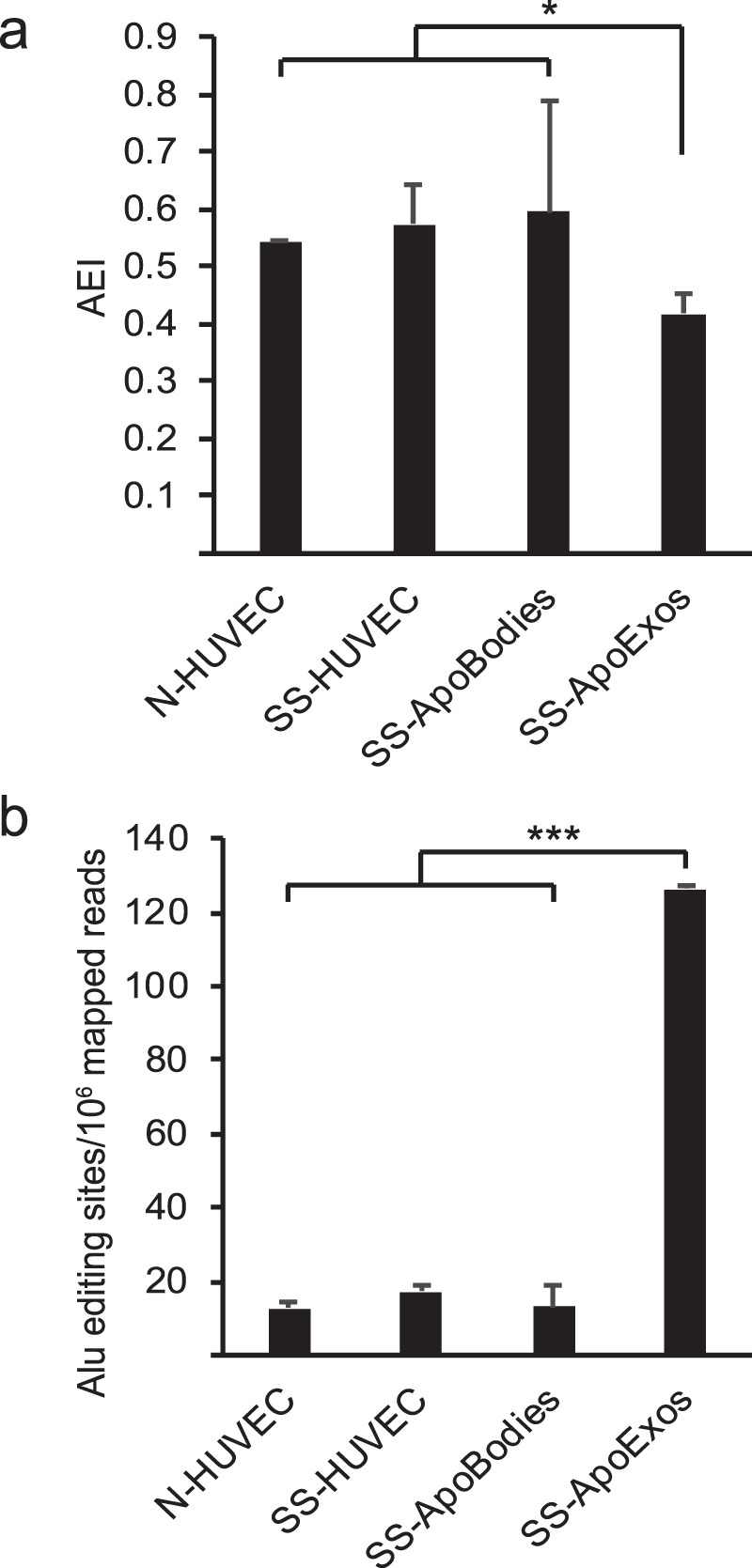
ApoExos contain large amounts of unedited *Alu* sequences. (**a**) *Alu* editing is reduced in ApoExos. AEI was calculated as the ratio of inosines to (adenosines + inosines) in positions identified by REDItools as real editing positions (coverage depth ≥10 reads, A to G mismatch frequency ≥1%, located within *Alu* regions) (*Two-tailed unpaired T test, p = 0.0498, n = 2). (**b**) ApoExos express 10 times more *Alu* editing sites. Number of *Alu* editing sites identified by REDItools across samples according to strict criteria (coverage depth ≥10 reads and A to G mismatch frequency ≥1%) (Two-tailed unpaired T test, ***p = 1.5 × 10^−3^, n = 2).

One type of SINEs enriched in ApoExos, *Alu* elements, occupy a unique place among EREs in view of their remarkable abundance and immunostimulatory properties. Having a copy number in excess of 1 million copies, these 300 base pairs sequences represent 11% of the human genome^42,43^. Moreover, upon transcription, inverted *Alu*-*Alu* sequences form cytoplasmic duplexes recognized by the MDA5 RLR^44^. In order to avoid MDA5-driven immunopathologies (e.g., Aicardi-Goutières syndrome), cells use adenosine deaminases acting on RNA (ADAR) to perform adenosine-to-inosine RNA editing and prevent the formation of RNA duplexes. More than 90% A to I RNA editing occurs in *Alu* regions, and its extent is most commonly evaluated using the Alu Editing Index (AEI)^43^. Employing REDItools^45^, we therefore analyzed *Alu* editing in STAR-mapped reads from HUVECs and EVs. After applying filters restricting the search to *Alu* regions covered by at least 10 reads and displaying at least 1% editing, we screened A to I (seen as G) nucleotide changes in all datasets. Two observations emerged from these analyses. First, the AEI was lower in ApoExos than in HUVECs and apoptotic bodies (Fig. 4a). Second, the total number of expressed (≥10 reads) *Alu* editing sites was increased by about tenfold in ApoExos relative to other datasets (Fig. 4b). Based on their decreased AEI and high abundance of *Alu* editing sites, we conclude that ApoExos contain large amounts of unedited *Alu* sequences poised to form duplexes and stimulate MDA5.

### HUVECs-derived ApoExos are enriched in A- and/or U-rich nucleotide motifs

**Figure 5:**
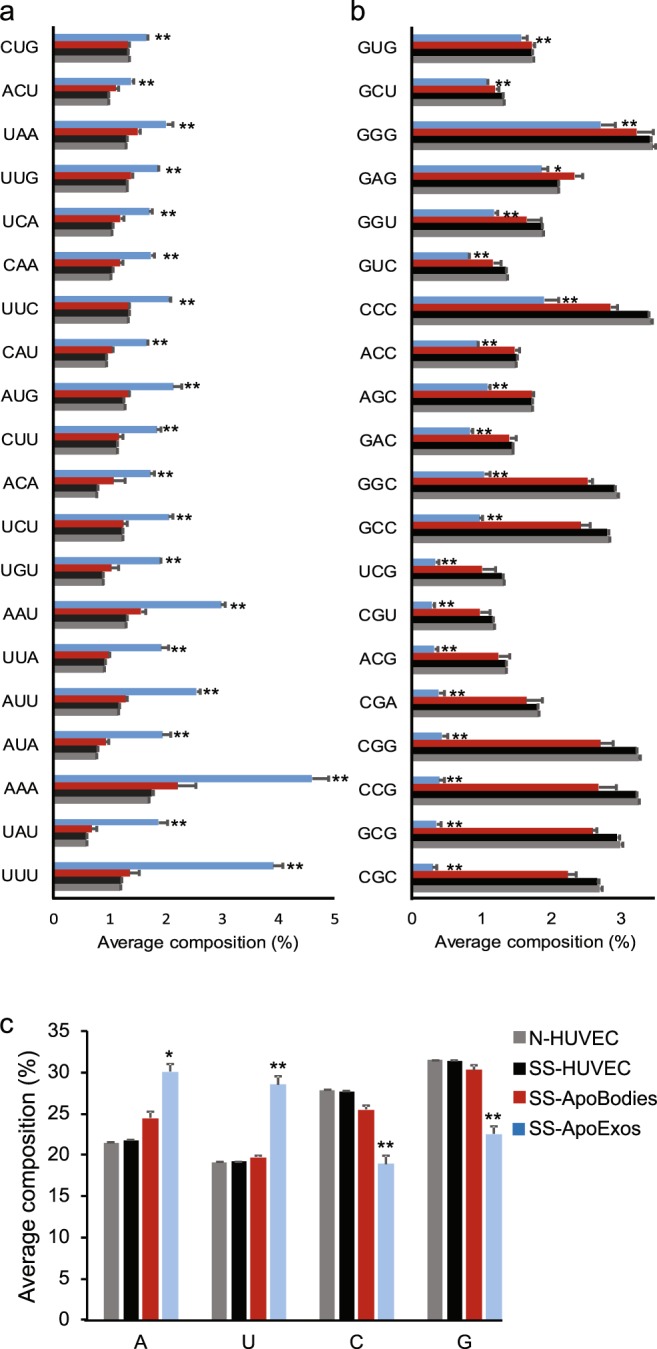
HUVECs-derived ApoExos are enriched in A- and/or U-rich nucleotide sequences (**a**,**b**) ApoExos contain more AU-rich (**a**) and less GC-rich 3nt motifs (**b**). Whole RNA sequencing adaptor-trimmed reads with good quality were chopped in 3nt-long k-mers which were then quantified using JellyFish. (**b**) Bar chart showing ApoExos top enriched motifs. (**b**) Bar chart showing motifs depleted in ApoExos. (**c**) ApoExos whole RNA load contains more uracil. Single nucleotide usage was calculated from same reads as in (**a**,**b**). (Two-tailed unpaired T test, *p ≤ 0.05, **p ≤ 0.01, n = 2).

To follow up on our global analyses of the genomic origin of RNAs enriched in ApoExos, we next sought to define the nucleotide composition of these RNAs. The rationale for this was that individual TLRs and RLRs preferentially recognize distinct RNA motifs. For example, poly-U and AU-rich sequences are preferred ligands of RIG-I and TLR8 while TLR7 is preferentially activated by poly-U and GU-rich sequences^46–50^. In order to evaluate whether RNAs from ApoExos displayed any bias in nucleotide usage, we performed a compositional analysis of RNA sequences by quantitating all trinucleotides (3nts) and pentanucleotides (5nts) from our datasets using Jellyfish^51^. These analyses clearly showed that ApoExos ribonucleotide content was very significantly enriched in A- and/or U-rich 3nts and 5nts (Fig. 5a and Supplemental Fig. 3a). Interestingly, significantly enriched sequences identified in ApoExos (AAA, UUU, AAU, CAA, UAA for 3nts and AAAAA, UUUUU for 5nts) correlated with immunostimulatory RNA sequences previously identified by Chaudhary *et al*. in their efforts to develop RNA-based vaccine adjuvants^52^. Consistent with the enrichment of A- and U-rich sequences, the abundance of G- and C-rich sequences was significantly decreased in ApoExos compared to apoptotic bodies and HUVECs (Fig. 5b and Supplemental Fig. 3b). Furthermore, single nucleotide usage was coherent with that of 3nts and 5nts sequences (Fig. 5c).

**Figure 6:**
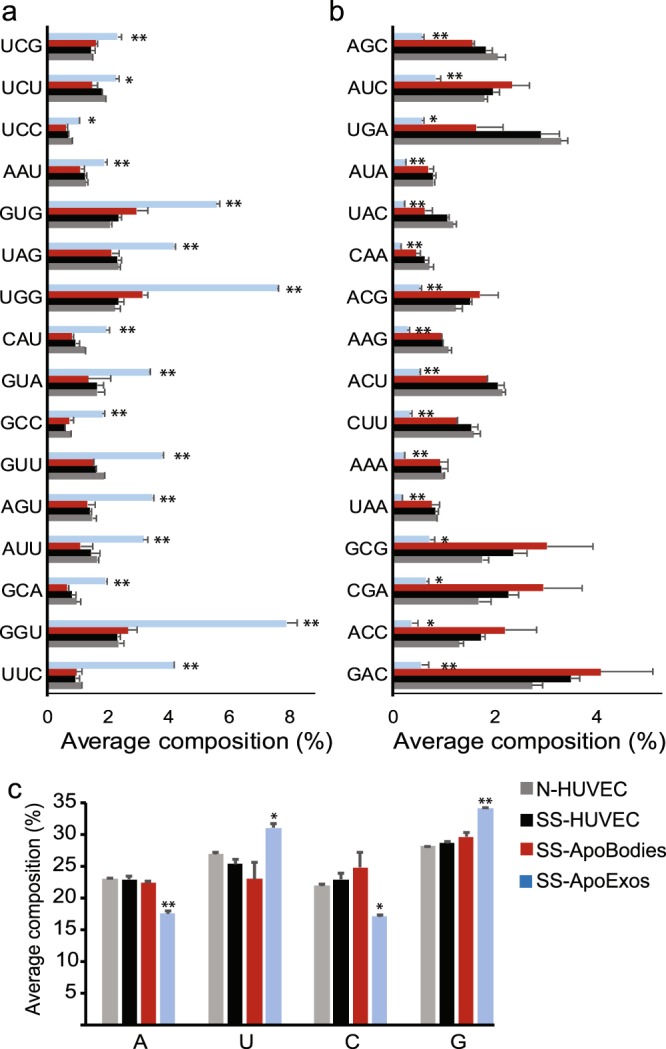
Small RNAs in HUVECs-derived ApoExos are enriched in U- and G-rich sequences. (**a**,**b**) ApoExos small RNAs contain more GU-rich (**a**) and less AC-rich 3nt motifs (**b**). Small RNA sequencing adaptor-trimmed reads with good quality were chopped in 3nt-long k-mers which were then quantified using JellyFish. (**a**) Bar chart showing ApoExos top enriched motifs. (**b**) Bar chart showing motifs depleted in ApoExos. (**c**) ApoExos small RNAs contain more uracil. Single nucleotide usage was calculated from same reads as in (**a**,**b**). (Two-tailed unpaired T test, *p ≤ 0.05, **p ≤ 0.01, n = 2).

The global analyses of RNA content depicted in Fig. 5 encompass both long and small RNAs. Because of their greater length, long RNAs have a dominant influence on the nucleotide content and might therefore overshadow features of small RNAs. To evaluate this possibility, we performed a compositional analysis selectively on small RNAs from small RNA sequencing data of the same samples. We found a clear enrichment in U- and G-rich, but not of A-rich, oligonucleotides in small RNAs from ApoExos relative to HUVECs and apoptotic bodies (Fig. 6a–c and Supplemental Fig. 4a,b). The most parsimonious explanation for the enrichment of A-rich sequences in total RNAs but not in small RNAs is that most small RNAs are not polyadenylated^53^. Overall, enrichment in U- and A-rich sequences in total RNAs, and of U- and GU-rich motifs in small RNAs endows ApoExos with a unique ability to stimulate various RLRs and endosomal TLRs^46–50^.

### HUVECs-derived ApoExos are enriched in unstable RNAs

**Figure 7:**
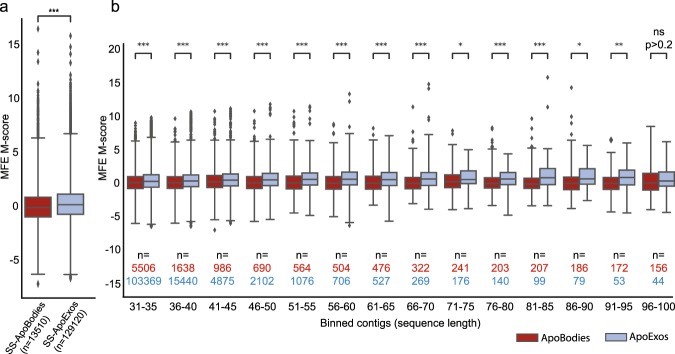
HUVECs-derived ApoExos present more dynamic RNAs. (**a**) Box plot depicting the distribution of MFE M-scores computed with MC-FlashFold for each RNA sequence identified as apoptotic bodies-enriched (ApoBodies) or ApoExos-enriched (ApoExos). All sequences (≤300nt) were included. (Two-tailed permutation test, p ≤ 0.001). (**b**) Box plots depicting the distribution of MFE M-scores (see Methods) computed with MC-FlashFold for each RNA sequence identified as apoptotic bodies-enriched (ApoBodies) or ApoExos-enriched (ApoExos), binned in intervals of 5 nucleotides. Since bins with sequences longer than 100 nucleotides had very low counts, they were not included in the plot. (Two-tailed permutation test, *p ≤ 0.05, **p ≤ 0.01, ***p ≤ 0.001).

Sensing by PRRs is regulated not only by the primary but also by the secondary structure of RNAs. Indeed, open structures of single stranded RNA, without stem loops, are preferentially recognized by TLR7 and TLR8 and are more immunostimulatory^52,54,55^. To gain insights into the secondary structure of ApoExos RNA sequences, we used MC-FlashFold^56,57^ to compute the minimum free energy (MFE) of random RNA sequences and that of RNAs with differential abundance in ApoExos vs. apoptotic bodies (see methods for details). A low MFE corresponds to a stably folded RNA sequence, whereas a high MFE is associated with more dynamic structures. A high-energy sequence is therefore more likely to exhibit secondary structure conformations that include open linear sequences. We selected MC-FlashFold for this analysis because of its unique ability to quantitatively assess the contribution of non-canonical base pairings in a sequence^56^, which enables accurate folding of RNA sequences that can be challenging to other predictive RNA folding algorithms. In our analyses, we excluded ribosomal RNAs because their secondary structure is molded by associated ribosomal proteins, and we strictly focused on sequences whose abundance differed by at least 4-fold in ApoExos vs. apoptotic bodies. Since MFE is dependent on sequence length, MFEs were normalized to MFE M- scores (modified Z-scores, see Methods). The notable finding was that globally, RNA sequences in ApoExos presented a higher MFE than in apoptotic bodies (Fig. 7a). The difference between apoptotic bodies and ApoExos was not caused by enrichment of RNAs of certain lengths in one type of EVs because this difference was still observed when RNAs of similar lengths were compared (Fig. 7b). Taken together, these results indicate that ApoExos contain more dynamic RNA structures which are more likely to exhibit linear sequences and thereby increase their immunostimulatory properties.

## Discussion

**Figure 8:**
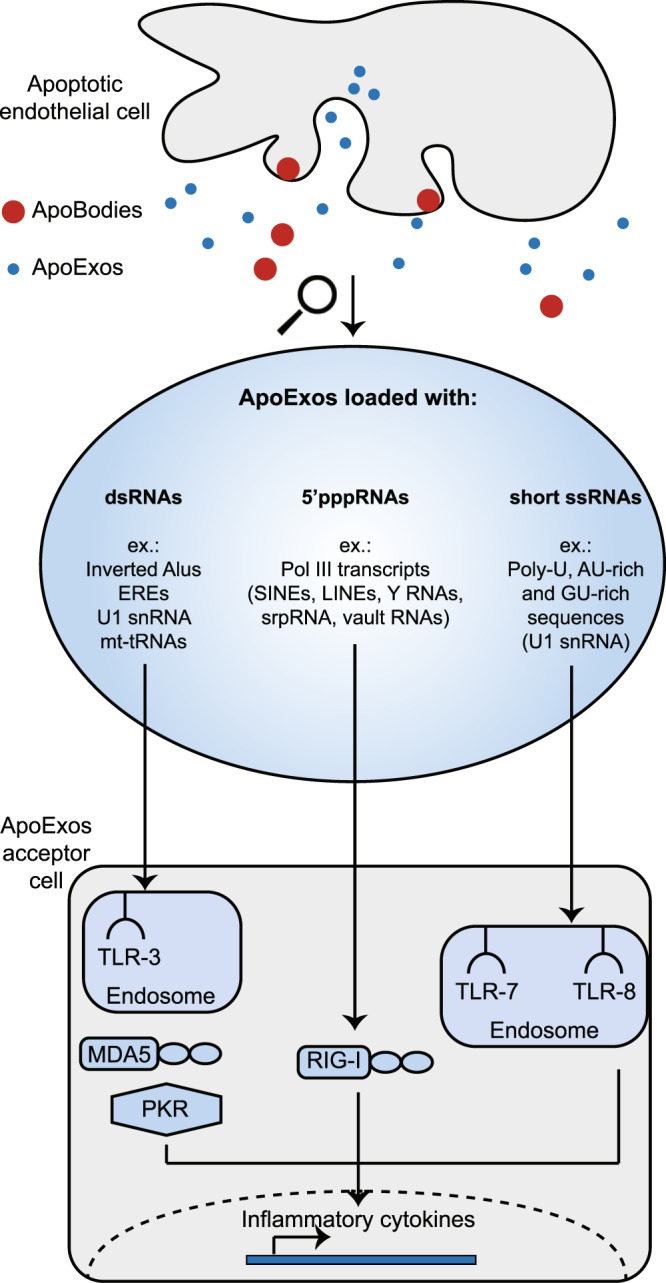
ApoExos are loaded with immunostimulatory RNAs. Schematic representation of RNAs enriched in ApoExos (Figs 2–6) and of their putative PRRs. mt-tRNA: mitochondrial transfer RNA, PKR: protein kinase R, snRNA: small nuclear RNA, srpRNA: signal recognition particle RNA.

At the organismal level, intercellular communication is vital and, in several conditions, is largely dependent on the ability of EVs to convey complex messages from transmitter to receiver cells^58^. This is particularly conspicuous in the case of chronic immune processes: responses to pathogens and cancer, inflammation, autoimmunity, graft rejection and GVHD^10,13,59,60^. Endothelial cells are ideally located to disseminate systemically the EVs that they secrete upon various stress conditions. *In vivo*, apoptotic endothelial cells release two types of well-characterized EVs upon caspase-3 activation: apoptotic bodies and ApoExos^14^. Only ApoExos are immunogenic: their injection causes inflammation and autoimmunity in mice^13^. The present work demonstrates that these two types of apoptotic endothelial cells-released EVs are loaded with divergent RNA cargos that are not released by healthy endothelial cells (Fig. 1). Apoptotic bodies, like HUVECs, contain mainly ribosomal RNA while ApoExos essentially contain non-ribosomal non-coding RNAs (Fig. 2). The non-coding RNAs in ApoExos are coded mainly by EREs and repetitive elements whose primary and secondary structure display typical features of RNAs recognized by a large variety of PRRs (summarized in Fig. 8). Of note, because ribosomal and non-coding RNAs are filtered out by standard RNA-Seq methods (e.g., poly-A capture and ribosome depletion)^22,24^, our unbiased strategy based on the sequencing of all RNAs was instrumental in uncovering the peculiar composition of EVs, and in particular their high content of long non-coding RNAs. Indeed, previous studies on the RNA content of EVs have largely focused on micro-RNAs, which represent only a minor fraction of RNAs found in ApoExos.

The transcriptome of ApoExos was unique in regard to its genomic origin, nucleotide composition and secondary structure. Indeed, as detailed below, all RNA families enriched in ApoExos have been shown to elicit innate immune responses in a large variety of experimental models (summarized in Supplemental Table 1). The most notable attribute of ApoExos is that they include all these RNA families and motifs in one single population of EVs. One striking feature is that 89% of RNAs in ApoExos were viral-like since they were coded by EREs and other repetitive elements (Fig. 3). Normally, these genomic sequences are silenced in somatic cells, mostly via DNA methylation. However, upon stress associated with carcinogenesis or autoimmunity^23^, they frequently undergo transcriptional activation. Expression of EREs is implicated in the pathogenesis of autoimmune diseases and has been shown to enhance the immunogenicity of cancer cells^23,35–37^. Indeed, ERE RNAs (including LINES, SINEs, and LTRs) were found to be enriched in exosomes released by cancer cells, to activate TLR3 and RLRs and to trigger IFN signaling^20,35,38,39,61^. EREs present in ApoExos can form double-stranded RNA and thereby stimulate TLR3 and MDA5 via two mechanisms^62^. First, EREs readily form RNA duplexes when they are transcribed from both DNA strands. Second, *Alu* sequences, a variety of SINEs, constitutively form RNA duplexes unless they are edited by ADAR enzymes. Since they contain huge quantities of EREs (Fig. 3) and unedited *Alu* sequences (Fig. 4), ApoExos are well equipped to stimulate TLR3 and MDA5.

Several non-coding RNAs enriched in ApoExos are notorious for their ability to stimulate PRRs. For example, the U1 RNA, which forms complexes with nuclear ribonucleoprotein (U1 RNP), has a specific role in autoimmunity. U1 RNA stimulates TLR3, TLR7, TLR8 and RIG-I, and autoantibodies against its associated nucleoproteins are present in all patients with mixed connective tissue disease^30–33^. Since uracil represents 26% of the U1 sequence (vs. 19% of the HUVEC transcriptome), U1 may contribute to the uracil enrichment in ApoExos (Fig. 5c). Of note, the presence of U1 RNA was previously reported in exosomes secreted by colorectal cancer cells^63^. A similar scenario takes place with Y RNAs, also highly represented in ApoExos (Fig. 2c,d) and other EVs^64^, which form complexes with Ro60 ribonucleoprotein: Y RNAs stimulate TLRs and the Ro60 ribonucleoprotein is recognized by autoantibodies in subjects with systemic lupus erythematosus^65^. Finally, the long non-coding signal recognition particle RNA RN7SL1, also enriched in ApoExos (Fig. 2b,d) and in EVs from stromal fibroblasts^66^, was shown to trigger RIG-I activation in breast cancer cells, promoting cancer progression and metastasis^66^.

The nucleotide composition and secondary structure of ApoExos were distinct from those of HUVECs and apoptotic bodies (Figs 5 and 6). Uracil usage was increased in ApoExos RNAs, consistent with the fact that endosomal TLR7 and TLR8, as well as cytoplasmic RIG-I, are preferentially stimulated by U-rich RNAs. Furthermore, a report from Salvi *et al*.^67^ demonstrated that exosomes isolated from the plasma of lupus erythematosus patients can activate endosomal TLR7 in plasmacytoid dendritic cells. This effect was reverted by chloroquine, indicating that endosomal acidification is required for dendritic cell activation. Salvi *et al*. also showed that this activation was due to G- and U-rich miRNAs present in the vesicles, coherent with the increased G- and U-nucleotide content found in our ApoExo small RNAs (Fig. 6). ApoExos where also enriched in unstable RNA structures, a feature that should further increase their ability to stimulate TLR7 and TLR8 (Fig. 7). This assumption is supported by a meta-analysis showing that a high MFE, a characteristic of dynamic RNA structures, was one of the best predictors of the ability of RNAs to trigger interferon production^68^.

We conclude that the RNA cargo of ApoExos is ideally suited to stimulate many PRRs (Fig. 8). These results provide a molecular framework for understanding the unique ability of ApoExos to trigger immune responses^13^. Indeed, they show that by selectively sorting and secreting immunostimulatory ‟self RNAs” into small EVs, apoptotic endothelial cells can lead to stimulation of immune responses causing graft rejection and autoimmunity. The fact that the RNA load of ApoExos can redundantly stimulate a large variety of RLRs and TLRs has one noteworthy implication for future studies: each PRR may be sufficient but none may be necessary to mediate the effects of ApoExos. Hence, deleting single PRRs in cells or animals is unlikely to abrogate immune response to ApoExos^69^. In addition, we speculate that non-ribosomal non-coding RNAs with the highest abundance in ApoExos (Fig. 2d) could potentially be used, after validation in patients presenting various medical conditions, as markers of endothelial damage. The mechanisms implicated in the sorting of non- ribosomal non-coding RNAs in ApoExos certainly represent an important area of future investigation. Notably, in line with the enrichment of polyU sequences in ApoExos (Figs 5 and 6), analyses in other models suggest that 3′ end uridylated isoforms are preferentially sorted in exosomes^70^. Another important issue that has to be addressed is the level of overlap/discrepancy in the RNA content of ApoExos released by different cell types exposed to various apoptosis inducers. On a more general note, data presented herein illustrate how unbiased systems-biology approaches can yield profound mechanistic insights in many fields such as immunology^71,72^.

## Methods

### Cell culture and isolation of EVs

Human Umbilical Vein Endothelial Cells (HUVECs, Sigma-Aldrich, 200P-05N) from two independent batches, each produced from a pool of 10 different individuals, were cultured in 175 cm^2^ flasks in endothelial-growth medium (Lonza). For EVs production, HUVECs were incubated in standard vesicles-free endothelial-growth medium or serum-free RPMI (Life Technologies) medium for 4 h. Vesicles-free medium was depleted from EVs by ultracentrifugation at 200,000 *g*, 4 °C for 18 h prior to cell culture. After 4 h of incubation, medium was collected and EVs were isolated through differential centrifugation as previously described^13^. Briefly, harvested medium was centrifuged for 15 min at 1200 g to remove apoptotic cells and cellular debris. Subsequently, the supernatant was collected and centrifuged for 15 min at 50,000 g to isolate apoptotic bodies. The remaining supernatant was collected and ultracentrifuged for 18 h at 200,000 g to pellet ApoExos. As reported previously, EVs isolated after centrifugation at 50,000 g are mostly apoptotic bodies ranging from 1 to 5 uM (containing intracytoplasmic components and various organelles, such as mitochondria) but also contain some smaller membrane vesicles within microvesicle size range (0.25 to 1 uM). The fraction isolated by centrifugation at 200,000 g is enriched in smaller EVs ranging in size from 30 to 100 nm. Isolated vesicles were resuspended in Trizol for RNA extraction or in PBS for use in functional assays.

### Quantification of exosome-like vesicles by flow cytometry

Quantification of HUVECs-derived exosome-like vesicles by flow cytometry was performed as previously described^13,73^. Briefly, HUVECs were stained with 5-chloromethylfluorescein diacetate (Thermo Fisher) according to manufacturer’s protocol. Cells were then cultured in standard vesicles-free or serum-free RPMI, as described in the previous section. Harvested EVs were analyzed with a FACS Canto II equipped with a forward scatter photomultiplier tube specific for detection of small particles. Briefly, during acquisition, vesicles are compared to fluorescent Sky Blue microspheres of 40–90 nm, 400–600 nm, 700–900 nm, 1000 nm and 2500–4500 nm diameter. An exosome-like (ApoExos) gate including particles from 100 to 1000 nm, and an apoptotic body gate including particles larger than 1000 nm in diameter were used to detect exosome-like vesicles and apoptotic bodies, respectively.

### RNA isolation, library construction and sequencing

Total RNA was extracted using TRIzol® Reagent (Life Technologies) according to the manufacturer’s protocol. RNA was then purified using the miRNeasy micro kit (Qiagen) and submitted to on-column DNAse I digestion using the RNAse-free DNAse set (Qiagen) as recommended. Samples quality and quantity were determined on Agilent 2100 Bioanalyzer using RNA 6000 Pico kit (Agilent Technologies). For whole transcriptome analysis, libraries were generated from 60 ng extracted RNA using the KAPA stranded RNA-seq kit (Roche) following the manufacturer’s protocols. Paired-end (2 × 80 base pairs) sequencing was performed using the Illumina NextSeq 550 system. Eight RNA-seq libraries (2 biological replicates of N-HUVECs, SS-HUVECs, apoptotic bodies and ApoExos) were sequenced in a single run yielding a total of 800 M reads (60 M to 120 M paired-end reads per sample). For small RNA sequencing, libraries from the same 4 duplicated samples were generated from 20 ng RNA using the CleanTag kit for small RNAs (TriLink). Single-read (1 × 75 base pairs) sequencing was performed on Illumina NextSeq 550 (50 M reads per sample).

### Analysis of reads distribution

The raw reads were trimmed to remove adapter sequences and low quality extremities (bases with quality values below 20) using Trimmomatic version 0.35. RNA-Seq data have been deposited in Gene Expression Omnibus archives under accession number GSE119108. For read distribution, reads were aligned on human genome GRCh38 (gene annotation from GENCODE version 26, based on Ensembl 88) using STAR version 2.5.1b. Mapped reads were then characterized as exonic, intronic or intergenic using RSeQC 2.6.3.

### Analysis of transcriptome and repetitive elements

For annotated transcripts analysis (Fig. 2), reads were quantified and aligned on Ensembl annotated transcripts (GRCh38.91) using Kallisto (v0.43.1)^26^. For global annotated transcripts and repetitive elements analysis (Fig. 3), a Kallisto index was built using Ensembl annotated transcripts (GRCh38.91, built from the gtf file using the gffread program) combined with all genomic repeats identifications (repeat masker database on GRCh38 from UCSC Table Browser). Transcripts expression levels were expressed as transcripts per million (TPM).

### Evaluation of RNA Editing Level

To identify RNA editing events within *Alu* sequences, we used REDItools^45^. STAR mapped RNA sequencing reads were submitted to the REDItoolDnaRna.py script detecting all single nucleotide mismatches between RNA-Seq data from each sample and GRCh38.88 reference genome. To limit the risk of false positives, we kept adenosine (A) to inosine (I) editing positions that were covered by at least 10 RNA sequencing reads and that presented an editing frequency ≥0.01. RNA editing positions located within *Alu* regions were then identified by intersecting genomic positions identified by REDItools and SINE table from repeat masker (UCSC Table Browser). AEI calculation was done as previously described^43^.

### Nucleotide patterns quantification

For total RNA sequencing data (Fig. 5), in order to work on stranded data, adapter-trimmed R1 reads were reverse complemented, and adapter-trimmed R2 reads were unmodified. For small RNAs (Fig. 6), all adapter-trimmed reads were used. We then, for both datasets, employed the k-mer counting tool JellyFish^51^ setting k-mer length to 3- or 5- nucleotides.

### RNA folding analysis

Adapter-trimmed paired-end reads from apoptotic bodies and SS-ApoExos were extended and merged using the BBMerge^74^ software. The resulting reads were quality trimmed with Cutadapt^75^ then stripped from their rRNA content by mapping to a rRNA transcript index using HISAT2^76^. Only unmapped reads were kept. Unmerged R1 reads were then reverse complemented and joined to their corresponding R2 reads with a gap of 40 uncalled nucleotides (N). The two resulting sets of reads (merged and joined) were chopped into 31-nt long sequences (k-mers) using Jellyfish ignoring unique k-mers. K-mer counts were normalized by considering read counts after rRNA removal. K-mers with a count lower than 10 in each sample were discarded. Differential k-mer occurrence analysis was performed using the software package edgeR^77^ in R^78^. A robust estimate of the negative binomial dispersion parameter was computed for each k-mer using the estimateGLMRobustDisp function^79^. A false discovery rate (FDR) cut-off of 5% was applied, keeping only statistically relevant k-mers that presented at minimum a 4-fold change between conditions. The dataset was separated in 2 smaller parts, each consisting of upregulated k-mers in each condition. The resulting 2 subsets of k-mers were assembled into longer sequences (contigs) separately using a de Bruijn graph assembler. All contigs ranging from 31 to 300 nucleotides were folded using MC-Flashfold^56^, returning an MFE value.

Because MFE values are only comparable between sequences of similar lengths, we proceeded to transform them into MFE modified z-scores (M-scores). For each RNA contig previously assembled, MFE values were transformed into M-scores as described by Iglewicz and Hoaglin^80^.

$${M}_{i}=\frac{({x}_{i}-{\tilde{x}}_{l})}{MA{D}_{l}},$$

where x̃ represents the median and MAD the Median Absolute Deviation of 100,000 randomly generated sequences of the same length *l* of contig *i*. The MAD value was calculated using the ‘mad’ function from the ‘statsmodels.robust.scale’ package of the Python programming language and using the default normalization constant value (approximately 0.6745). This transformation was chosen because of its property to center the median around 0 for a given distribution, making comparisons more straightforward in cases of non-normality.

### Permutation test method

To assess the statistical significance of the observed energy differences between samples, a permutation test was applied to the MFE M-scores and the binned MFE M-scores. A two-sided permutation test was performed to determine whether the MFEs in ApoExos are higher than the ones in apoptotic bodies. First, the median difference of the measures was obtained, then the two conditions were pooled together in 100,000 randomly chosen ways of dividing the data, in sets mimicking the respective size of the original samples. The p-value was defined as the proportion of the median differences for each of the randomly generated samples, whose value was more than or equal to the observed median of the original sets. If the p-value was less than 0.05, the null hypothesis that both samples come from the same distribution of energies was rejected and the alternative hypothesis that ApoExos RNAs and apoptotic bodies RNAs have a differing general tendency in folding was accepted.

## Supplementary information

Supplementary Information
