# Supplementary material for: Apoptotic endothelial cells release small extracellular vesicles loaded with immunostimulatory viral-like RNAs
Source: Sci Rep. 2019 May 10;9:7203. doi: 10.1038/s41598-019-43591-y (PMC6510763; doi:10.1038/s41598-019-43591-y)
Supplement: Supplementary file 1 — Supplementary Information [file 41598_2019_43591_MOESM1_ESM.pdf]

Supplementary information

Apoptotic endothelial cells release small extracellular vesicles loaded with immunostimulatory viral-like RNAs

Marie-Pierre Hardy[1,2], Éric Audemard[1], Francis Migneault[2,3], Albert Feghaly[1], Sylvie Brochu[1,2], Patrick Gendron[1], Éric Boilard[2,4], François Major[1,5,6], Mélanie Dieudé[2,3], Marie-Josée Hébert[2,3,7], Claude Perreault[1,2,7*].

[1]Institute for Research in Immunology and Cancer, Université de Montréal, Montreal, QC, Canada, H3C 3J7.
[2]Canadian National Transplant Research Program, Edmonton, Alberta, Canada, T6G 2E1.
[3]Research Centre, Centre Hospitalier de l'Université de Montréal (CRCHUM), Montreal, QC, Canada, H2X 0A9.
[4]Centre de Recherche du Centre Hospitalier Universitaire de Québec, Faculté de Médecine de l'Université Laval, Québec, Québec, Canada
[5]Department of Computer Science and Operations Research, Université de Montréal, Montreal, QC, Canada, H3C 3J7.
[6]Department of Biochemistry, Faculty of Medicine, Université de Montréal, Montreal, QC, Canada, H3C 3J7.
[7]Department of Medicine, Université de Montréal, Montreal, QC, Canada, H3C 3J7.

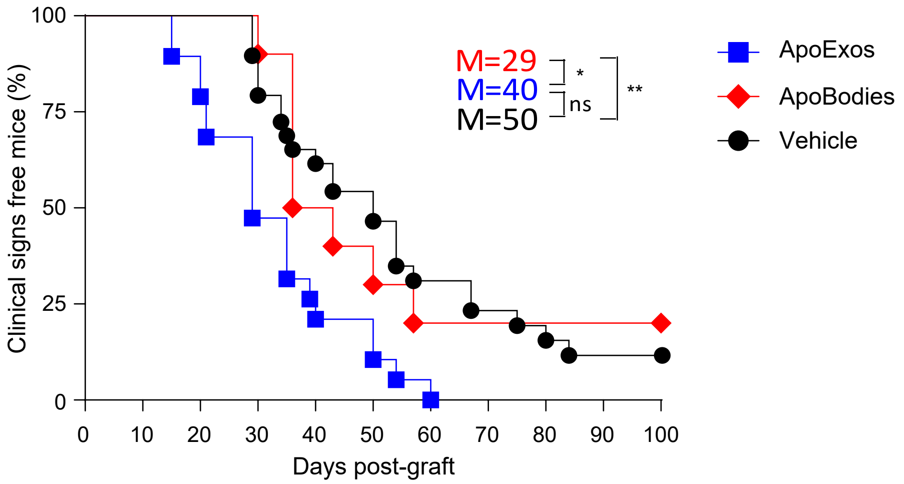

Supplemental figure 1. ApoExos increase GVHD severity. Lethally-irradiated (950cGy) recipient B6.129F1 mice were grafted with $10x10^6$ bone marrow cells and $60x10^6$ of a mix of spleen and lymph node cells from B6.SJL. Purified ApoExos (solid blue line) or ApoBodies (solid red line) collected from 0.45ml of serum-starved murine C57BL/6 endothelial cells supernatant (Dieudé M. *et al*; Science Translational Medicine; 2015 Dec 16; 7(318):318ra200) or equal volume of PBS (solid black line) were iv injected on day 0,+2,+4,+6 and +8 post-graft. GVHD clinical signs (weight loss, fur texture, posture, activity and skin integrity) were then monitored over time until day +100 using GVHD score as a metrics (Cooke K.R. *et al*; Blood; 1996 Oct 15; 88(8):3230-9). M represents the median of GVHD signs (GVHD score ≥2) appearance time. The log-rank test was used to compare the time of GVHD clinical signs appearance between the 3 groups. ApoExos: n=19, ApoBodies: n=10, PBS: n=30. *p≤0,05; ** p≤0,01.

a

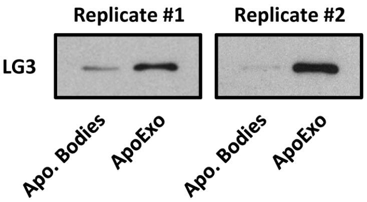

b

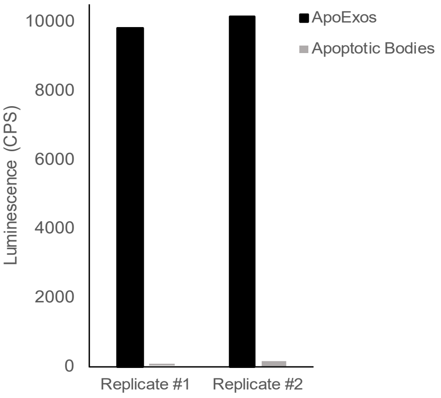

Supplemental figure 2. HUVECs-derived ApoExos contain elevated levels of LG3 and high caspase-like proteasome activity. (a) Immunoblot of LG3 in ApoExos or apoptotic bodies purified from the 2 replicatess of serum-starved apoptotic HUVECs used for transcriptomic analyses. The antibody used for Western blotting was an anti-perlecan from Santa Cruz Biotechnology. (b) Quantification of proteasome caspase-like proteolytic activity in ApoExos (black bars) and apoptotic bodies (grey bars) purified from the 2 replicates of serum-starved apoptotic HUVECs used for transcriptomic analyses. The assay was performed in white 96-well plates using the Proteasome-Glo Cell-Based Assay (Promega) according to the manufacturer's instructions using ApoExos (0.4 µg) or apoptotic bodies (0.4 µg) from serum-starved HUVECs supernatant. Luminescence was measured using a PerkinElmer Victor 3 V 1420 Multilabel Counter 1420-040 Microplate Reader.

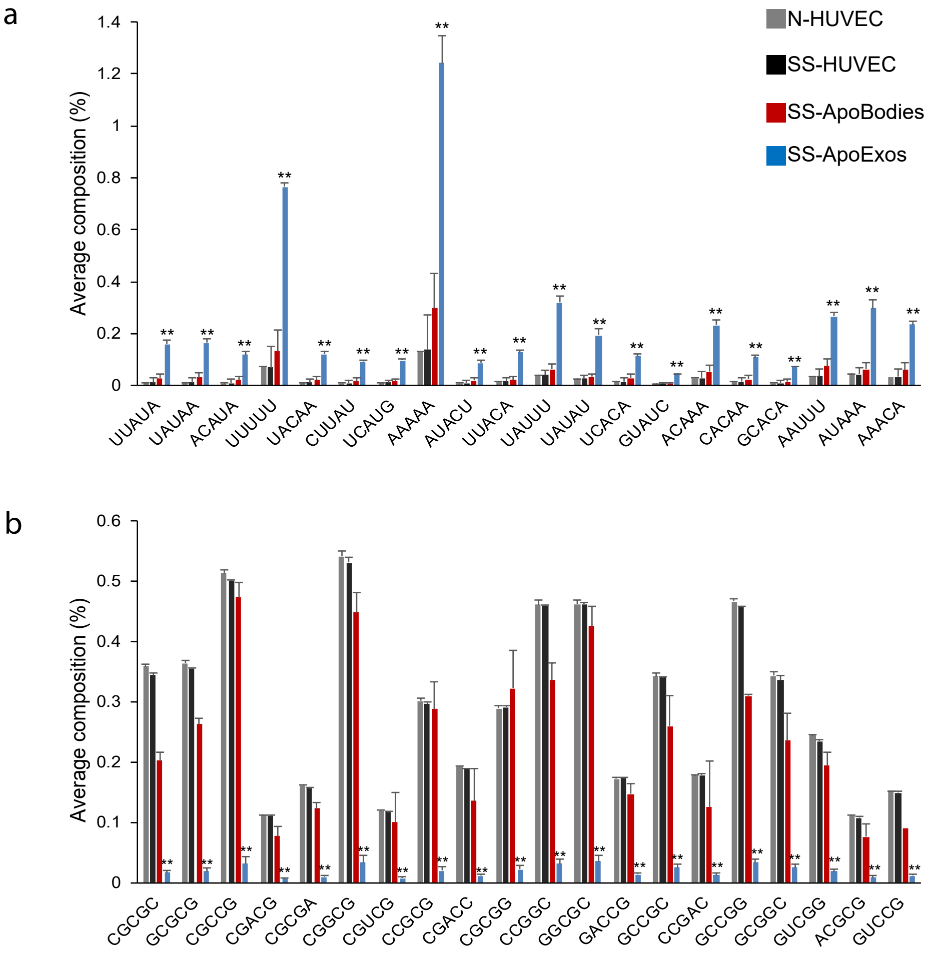

Supplemental figure 3. HUVECs-derived ApoExos RNAs contain more A and/or U-rich and less G and/or C-rich 5nt motifs. Whole RNA sequencing adaptor-trimmed reads with good quality were chopped in 5nt-long k-mers which were then quantified using JellyFish. (a) Bar chart showing ApoExos top enriched motifs. (b) Bar chart showing ApoExos most repressed motifs. (Two-tailed unpaired T tests, *p≤0.05, **p≤0.01, n=2).

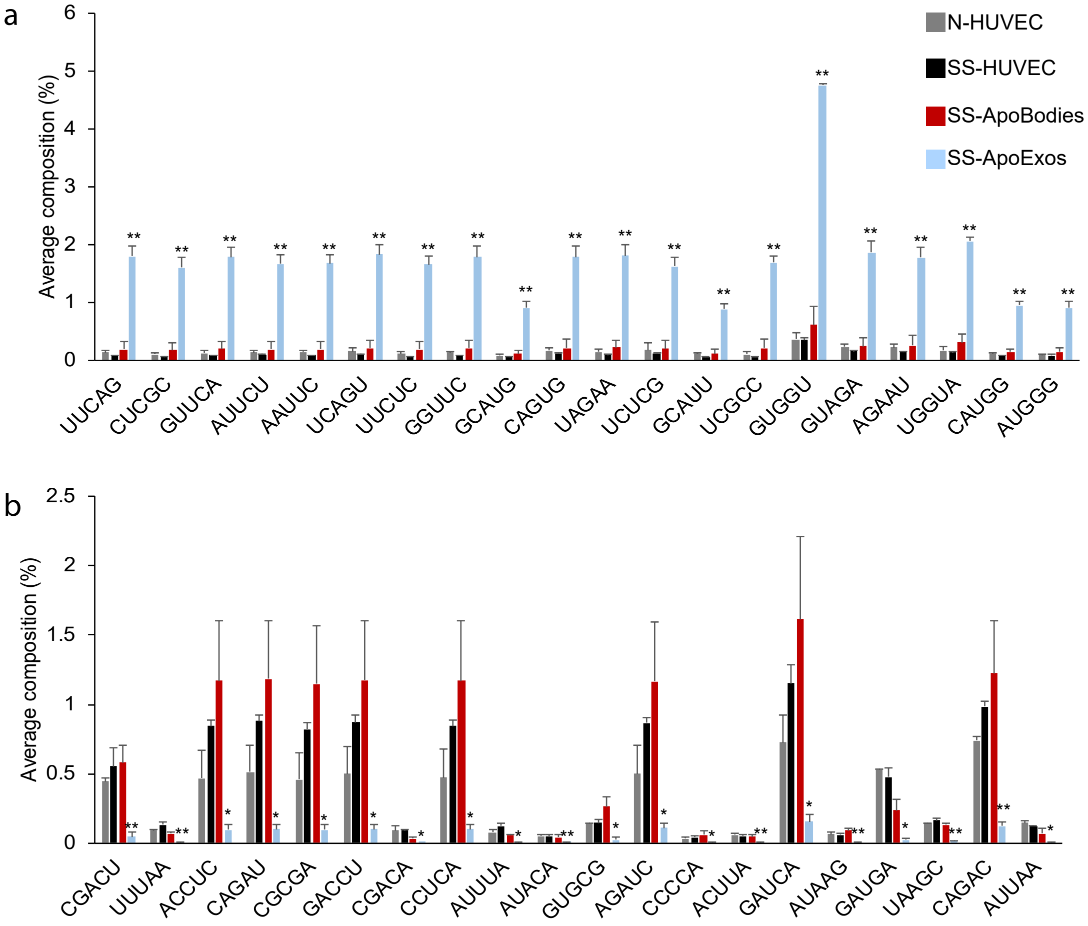

Supplemental figure 4. HUVECs-derived ApoExos small RNAs contain more G and/or U-rich and less A and/or C-rich 5-nt motifs. Small RNA sequencing adaptor-trimmed reads with good quality were chopped in 5nt-long k-mers which were then quantified using JellyFish. (a) Bar chart showing ApoExos top enriched motifs. (b) Bar chart showing ApoExos most repressed motifs. (Two-tailed unpaired T tests, *p≤0.05, **p≤0.01, n=2).

RNA family or feature enriched in ApoExos	PRR(s) stimulation	Involvement in immune response	References
U1 snRNA	TLR3, TLR7, TLR8, RIG-I	Triggers production of autoantibodies against snRNP	30-33
miRNAs	TLR7, TLR8		28, 67
mt-tRNAs	PKR		29
Y RNAs	TLR3, TLR7	Trigger production of autoantibodies against Ro60	64-65
EREs (LINE, SINE, LTR)	TLR3, RIG-I, MDA5	Trigger IFN signaling, enhance the immunogenicity of cancer cells	20, 23, 35-40, 61
5’PPP motif<br>(RNA polymerase III transcripts (ex. vault RNAs, RN7SL1/2, EREs))	RIG-I		20, 66
dsRNAs (ex. LTRs, inverted Alu-Alu duplexes)	TLR3, MDA5	Promote autoimmunity (Aicardi-Gouttières syndrome)	23, 35-40, 44
U-rich, AU-rich and GU-rich sequences	TLR7 and/or TLR8		46-50, 52, 67
Linear RNA structures	TLR7, TLR8		52, 68

Supplemental Table I. Immunostimulatory attributes of ApoExos-enriched RNAs. miRNA: microRNA, mt-tRNA: mitochondrial transfer RNA, snRNA: small nuclear RNA, srpRNA: signal recognition particle RNA, ERE: endogenous retroelements.
